# Enhancing Reliability of Studies on Single Filament Memristive Switching via an Unconventional cAFM Approach

**DOI:** 10.3390/nano11020265

**Published:** 2021-01-20

**Authors:** Niko Carstens, Alexander Vahl, Ole Gronenberg, Thomas Strunskus, Lorenz Kienle, Franz Faupel, Abdou Hassanien

**Affiliations:** 1 Chair for Multicomponent Materials, Institute for Materials Science, Faculty of Engineering, Christian-Albrechts-University of Kiel, Kaiserstraße 2, D-24143 Kiel, Germany; nic@tf.uni-kiel.de (N.C.); alva@tf.uni-kiel.de (A.V.); ts@tf.uni-kiel.de (T.S.); 2 Chair for Synthesis and Real Structure, Institute for Materials Science, Faculty of Engineering, Christian-Albrechts-University of Kiel, Kaiserstraße 2, D-24143 Kiel, Germany; og@tf.uni-kiel.de (O.G.); lk@tf.uni-kiel.de (L.K.); 3 Department of Condensed Matter Physics, J. Stefan Institute, Jamova 39, 1000 Ljubljana, Slovenia

**Keywords:** neuromorphic engineering, cAFM, diffusive memristive switching, resistive switching, memristors, spiking dynamics

## Abstract

Memristive devices are highly promising for implementing neuromorphic functionalities in future electronic hardware, and direct insights into memristive phenomena on the nanoscale are of fundamental importance to reaching this. Conductive atomic force microscopy (cAFM) has proven to be an essential tool for probing memristive action locally on the nanoscale, but the significance of the acquired data frequently suffers from the nonlocality associated with the thermal drift of the tip in ambient conditions. Furthermore, comparative studies of different configurations of filamentary devices have proven to be difficult, because of an immanent variability of the filament properties between different devices. Herein, these problems are addressed by constraining the memristive action directly at the apex of the probe through functionalization of a cAFM tip with an archetypical memristive stack, which is comprised of Ag/Si_3_N_4_. The design of such functionalized cantilevers (entitled here as “memtips”) allowed the capture of the long-term intrinsic current response, identifying temporal correlations between switching events, and observing emerging spiking dynamics directly at the nanoscale. Utilization of an identical memtip for measurements on different counter electrodes made it possible to directly compare the impact of different device configurations on the switching behavior of the same filament. Such an analytical approach in ambient conditions will pave the way towards a deeper understanding of filamentary switching phenomena on the nanoscale.

## 1. Introduction

The research field of memristive switching phenomena has already experienced huge interest for more than a decade. An important stimulus to this field came in 2008 [1], where for the first time a physically implemented resistive switching device was related to the originally proposed theory postulated in 1971, in which the memristor was described as the missing, fourth fundamental circuit element [2]. Despite the arising controversy following this claim [3], the application potential of memristive devices in technologies like in neuromorphic computation architectures [4,5,6], novel data storage [7], and memsensing [8,9] is unambiguous. Typically, memristors are two-terminal devices, which are capable of occupying different distinct resistive states, resulting from stimulation by an external electrical field. This functionality emulates certain aspects of the behavior of biological synapses, and makes memristors promising candidates for fundamental building units in brain-inspired hardware design [10]. Up to now a broad variety of physical mechanisms have been explored, which allow for the realization of memristive switching functionality, of which the electro-chemical metallization (ECM), the valence change mechanism (VCM), and the phase change mechanism (PCM) are the most prominent [11,12]. Among these different mechanisms, special attention has been paid to the ECM concept, which relies on an electrochemical redox reaction and migration of active metallic species, like Ag or Cu, between two electrodes. The application of controlled voltage stimuli on an ECM device allows the modification of the atomic configuration of the active species between the electrodes, and therefore influencing the resistive state, depending on whether or not both electrodes are electrically connected via a metallic path [13]. Taking the ECM mechanism as a fundamental working principle, memristive devices with a broad range of characteristics and different operation regimes have been created, like bipolar switching, complementary switching, or multilevel switching [14]. Special interest has been paid to memristive devices showing a diffusive switching characteristic, i.e., devices with very short filament lifetimes leading to volatility of the conductive state at low voltages. Diffusive memristive switching has been adapted to emulate short-term plasticity in neural systems [15], for the implementation of random number generators [16], or for selector devices [17]. The principal approaches for achieving diffusive switching are effectively limiting the amount of active species, e.g., by using nanoparticles as a reservoir for active species [18], or through severe current limitation during the filament formation process, e.g., by applying a current compliance, or by inclusion of a serial resistance [19].

A deep understanding of memristive switching phenomena is essential to reach the next levels in device maturity. Since investigating memristive features down to the nanoscale is inevitable, the exploitation of reliable and non-invasive analytical methods is challenging. In this regard, conductive atomic force microscopy (cAFM) is an established analytical tool, because it enables monitoring the electrical properties by a nano-sized probe at the required scale [20,21], and allows drawing conclusions between the measured electrical data and the morphology of the conducting filament [18,22,23]. Nevertheless, several prospects for cAFM, like studying the possible failure mechanism from endurance tests, are restricted by thermal drift effects, such as a spatial drift between the conducting filament and probe, leading to a considerable loss in data significance. Another challenge arises from the fact that measurements on different samples, representing the same filamentary device, might not be comparable to each other, because the switching performance of an individual filament considerably depends on the initial forming conditions and the matrix defect structure surrounding the filament [24]. Hence, complications exist when the influence of a parameter, like various counter electrode materials, must be studied over a sample series consisting of different devices, due to the inherent randomness of the filament switching performance. This problem has been encountered in many studies on memristive devices, and is commonly referred to as device-to-device variability [25,26]. Consequently, unconventional cAFM approaches are required to cope with the aforementioned problems. A promising strategy is to use cantilevers, which are functionalized with the memristive materials of interest. Instead of contacting the sample with a regular conducting cantilever, like in conventional cAFM, direct integration of the memristive thin film stack of interest on the apex of the cantilever is a versatile and unelaborate strategy to circumvent challenges like thermal drift, and to enable studies of different device configurations with the same active filament. Such an unconventional cAFM approach has been successfully used for a detailed characterization of memristive TiO_2_-based systems, with a focus on the chemical bonding status at interfaces in the device [27].

**Figure 1:**
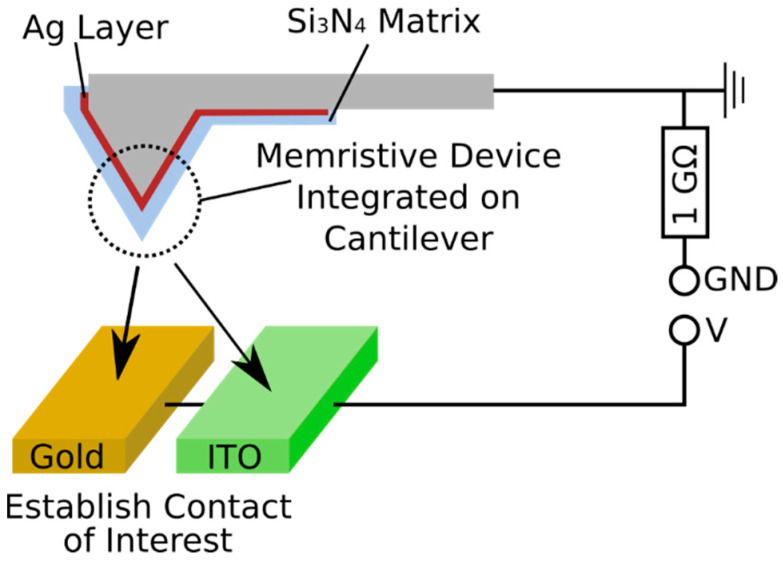
Schematic of the memtip approach. The memristive device, consisting of an Ag layer and an overlaying Si_3_N_4_ layer is integrated directly on the tip of the cantilever. By using conductive atomic force microscopy (cAFM) instrumentation, a reliable contact between the memtip and counter electrode of interest (which are Au and ITO in this work) is established and characterized by electrical measurements. A serial resistance of 1 GΩ was added in each measurement to limit the current through the memtip.

This work adapts this analytical approach towards ECM systems, which substantially extend the capabilities of cAFM for the investigation of memristive switching phenomena on the nanoscale. To demonstrate this, commercially available cantilevers were functionalized with a representative memristive device consisting of a stack of an active Ag layer and a Si_3_N_4_ dielectric layer. We note that there are other memristive systems which are also promising for future large scale device integration. The focus of our work, however, is to demonstrate pathways for reliable studies of single filament switching, for basic research purposes. Electrical characterization was done by contacting the functionalized cantilevers to planar counter electrodes, and measuring the IV-curve via a conventional cAFM instrument. In the following, the functionalized cantilever samples are referred to concisely as “memtips”. Figure 1 illustrates the approach of using memtips for enhanced cAFM studies. It should be noted, that the memtips could be applied principally to any conducting surface, and therefore testing of a variety of application scenarios is possible. The results presented in this work give fundamental insights into the diffusive switching of ECM devices, and are discussed in the individual sections under elucidation of the decisive advantages of the memtip approach. First, observations from a long-term measurement of a memtip are discussed, with a focus on temporal correlations between subsequent switching events. The focus on temporal correlations is motivated by the fact that encoding information in temporal patterns of memristor activity is seen as the next step in the development of artificial neural network hardware. Utilization of a memtip for the long-term observation turned out to be highly beneficial, because the filament formation was constrained to the vicinity of the apex and, accordingly, no drift between filament and apex was possible. This work further demonstrates a study on the influence of two different counter electrodes, namely Au and indium tin oxide (ITO), on the switching performance of the same active filament. This was enabled by the memtip approach, because here an identical memtip can be contacted to different counter electrodes. The last part of this work is dedicated to the occurrence of highly dynamic transitions between different resistive states, leading to distinct spiking patterns, which inherently arise when ECM devices are operated at the border of filament stability. Fundamentally, the characterizations done by the memtip approach resemble those of conventional cAFM on similar memristive devices. Principally, every conventional cAFM study on any memristive device could be converted to the memtip approach. The chosen approach of integrating the memristive stack directly on the cAFM tip mitigates thermal drift effects, and allows the study of the same active filament in different device configurations, ultimately leading towards an enhanced level of reliability and significance.

## 2. Materials and Methods

### 2.1. Preparation of Memtips

The fabrication of memtips was realized by covering commercially available cantilevers (Bruker OTESPA-R3, Camarillo, CA, USA) with an inert conducting layer over the entire surface, followed by integration of a memristive device directly on the apex on the cantilever by different physical vapor deposition methods in a self-built high vacuum deposition system. In the first vacuum deposition step, each cantilever was covered with a Ti layer acting as an adhesion promoter, followed by covering with an Au layer acting as a chemically inert electrode to make the memtip applicable for cAFM. In the second vacuum deposition step, a Ag layer and Si_3_N_4_ layer on top were deposited onto the apex of the cantilever under shadowing of the cantilever body, to integrate the memristor functionality.

The deposition system consisted of a turbo molecular pump (Pfeiffer Vacuum, TMU 262, Asslar, Germany) combined with a dry scroll pump (Agilent Technologies, SH-110, Santa Clara, CA, USA), to realize high vacuum conditions. Two independent DC magnetron sources (Thin Film Consulting, ION’X-2UHV, Grafenberg, Germany) connected to a high voltage power supply (Advanced Energy, MDX 500, Frankfurt, Germany) and mounted to the system were used for deposition of the corresponding layers in each step. The angle of incidence of each magnetron source with respect to the sample holder was 55°. Two flow controllers (Pfeiffer, EVR116 with attached hot ion cathode IMR 285, Asslar, Germany) regulated the inlet of inert Ar gas and reactive N_2_ gas for the according depositions. The sample holder in the deposition chamber was enabled for 360° rotations, which allowed uniform coating of the memtip from all sides, where it was needed.

Deposition of the Ti adhesion promoter was done by DC magnetron sputtering at 30 W and an Ar flow of 50 sccm from a Ti target. The subsequent Au deposition was done by DC magnetron sputtering at 30 W and an Ar flow of 50 sccm from an Au target. During both Ti and Au deposition steps, the cantilever was mounted vertically on the sample holder, and performed a complete 360° rotation over the deposition time, to ensure coverage of the entire surface. Deposition of the Ag layer was done by DC magnetron sputtering at 30 W and an Ar flow of 50 sccm from an Ag target. The final Si_3_N_4_ deposition was done by DC reactive sputtering from a Si target, at a power of 20 W and under a gas mixture of 50 sccm Ar and 0.44 sccm N_2_. The memtip was positioned in front of the magnetron source during Ag and Si_3_N_4_ deposition, to ensure vertical incidence of sputtered material to achieve homogeneous deposition in the apex region.

The memtips discussed in Section 3.1 and Section 3.2 were prepared with effective thicknesses of 3.5 nm for Ag and 4 nm for Si_3_N_4_. To realize the investigations on filament instability related to a limitation of active Ag species, the memtips discussed in Section 3.3 were prepared with effective thicknesses of 2 nm for Ag, and 6 nm for Si_3_N_4_.

### 2.2. Conductive AFM Instrumentation

The electrical measurement was carried out at room temperature using a commercial AFM microscope (Dimension 3000, Bruker, Camarillo, CA, USA). The electrical signals to the AFM were customized, using a break box, for device initialization and subsequent data acquisition. Non-invasive measurements were achieved with a contact force of 1.2 nN. The tip quality was also checked before and after measurements, to ensure that the tip apex suffered no degradation during prolonged measurements. The details of the measurements are described elsewhere [18].

### 2.3. TEM Investigation

TEM measurements were performed using a JEOL-2100 (Tokyo, Japan) with acceleration voltage of 200 kV and a LaB6 cathode. The memtip was mounted on a high-tilting holder, and tilted to 60° to almost obtain a sideview of the tip.

## 3. Results and Discussion

### 3.1. Long-Term Observation of Nanoscale Filamentary Switching

Results from long-term studies acquired by AFM methods frequently suffer from thermal drift effects, since they cause uncertainties with regard to the cantilever position relative to the feature under investigation. The lateral motion of the cantilever due to thermal drift is more than 10 nm h^−1^ making reliable long-term observation of nanoscalar features on the same order challenging [28]. As many memristive phenomena, especially those where filament formation is involved, take place in a region of several nm, conventional cAFM is not a reliable tool for long-term observation. This drawback of cAFM motivates the application of memtips for the study of filamentary-based memristive devices. Integrating the memristive device on the cantilever constrains the filament formation to occurring directly at the apex, and consequently prevents that filament and cantilever drifting apart from each other.

**Figure 2:**
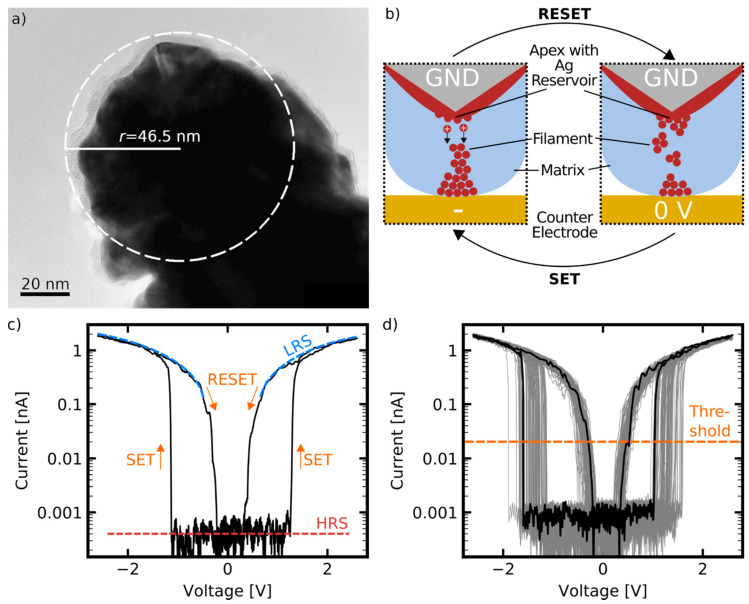
(**a**) TEM image of a memtip indicating a radius of curvature of 46.5 nm. (**b**) Schematic representation of the switching mechanism from electro-chemical metallization (ECM) principles. Upon application of an electrical field between the memtip and counter electrode, electrochemical oxidation of Ag is induced at positive polarity, leading to release and migration of Ag+-cations through the dielectric layer towards the negative polarity. Reduction of Ag+-cations occurs at negative polarity, causing the buildup of a conducting Ag filament, and leading to a SET switching event. Reduction of the electrical field causes a spontaneous disintegration of the filament because of interface energy minimization, resulting in a RESET switching event. (**c**) Representation of a single cycle from cAFM operation depicts the diffusive memristive switching characteristic of the memtip. The current in the low resistive state (LRS) is limited by the 1 GΩ serial resistor incorporated in the experimental setup. The high resistance state (HRS) is given by the detection limit of the setup. (**d**) Representation of 100 sequential cycles showing a consistent switching window. A threshold level of 20 pA, as indicated, is chosen for a reliable detection of SET and RESET.

In this section, the capabilities of memtips for the long-term observation of nanoscale memristive phenomena under the significant mitigation of thermal drift effects are demonstrated. Figure 2a depicts the apex of a memtip investigated by transmission electron microscopy (TEM). For this purpose, the entire cantilever was examined in the TEM in projection onto the side view. It can be seen, that the active area of the cantilever is uniformly covered with the Si_3_N_4_ layer. The dielectric layer has a thickness in the range of 4 nm ±1 nm (cf. Supporting Information Figure S1). We noted that the thickness variation of Si_3_N_4_ and the non-flat contour of the tip would be exclusion criteria for their applicability as electronic devices. However, for basic research into the switching activity of a single filament, these influences are secondary. From the TEM image the radius of the curvature can be estimated as 46.5 nm. The effective radius of curvature at the apex exceeds the specifications of the manufacturer due to the fabrication procedure of the memtip with additional Au, Ag, and Si_3_N_4_ layers. However, the thin coating ensures that the apex is still sharp enough to image features of less than 3 nm (cf. Supporting Information Figure S2). Due to beam damage, the cantilever examined in the TEM did not show diffusive switching, which was most likely based on a depletion of Ag atoms at the apex location. Those morphological changes also makes TEM studies on operated memtips critical. Further details can be found in the Supporting Information Figure S3, where morphological changes are illustrated. Electrical studies of the memtips were conducted by mounting the memtip into a conventional cAFM instrument, and adjusting the contact to an Au counter electrode. For the long-term study the memtip was operated continuously for roughly 18 h. The resulting electrical characteristics showed reliable diffusive memristive switching for over 12,000 cycles, with about 95% of all cycles showing a clear diffusive switching fingerprint. In the residual 5% that were characterized as no clear diffusive switching, no switching events, a nearly ohmic behavior, or a negligible switching window below 0.2 V were observed. The underlying physical mechanisms, together with a representative cycle, are depicted in Figure 2b,c, respectively. Under initial conditions, with no external field applied, the memtip is in a high resistance state (HRS) with a current level of 1 pA corresponding only to noise. Upon the application of an external electrical field, i.e., by ramping up the voltage towards either polarity, electrochemical oxidation of Ag takes place at anodic sites in the memtip, leading to the release of Ag+-cations into the dielectric layer. The Ag+-cations migrate along the field gradient until they become reduced at cathodic sites. By this mechanism Ag is removed from anodic sites, and accumulated at cathodic sites until a continuous filament has grown up and connects the cantilever apex with the counter electrode. The moment of completed filament formation is denoted as a SET event, and causes the transition to a low resistive state (LRS). In the LRS a current in the order of 1 nA flows through the memtip. Here, the resistance is not dominated by the memtip anymore, but by the 1 GΩ serial resistance, which is incorporated in the experimental setup. Subsequent reduction of the external field causes a spontaneous disintegration of the filament before reaching the zero-crossing, and accordingly results in a transition back into the HRS. This moment is denoted as a RESET event. As this mechanism takes place equally at both polarities, a full cycle is governed by four threshold switching events, namely one SET and one RESET event for each, positive or negative, bias voltage polarity. The fact that the LRS is not maintained at zero volt conditions is the most prominent feature of diffusive switching devices, and discriminates them from bipolar or unipolar memristive devices. A possible origin of this diffusive switching behavior are the short filament lifetimes, arising from the small filament diameters. If the formed filament is not thick enough, the overall energy of the system can be minimized by disintegration of the filament into individual clusters because of the high surface-to-volume ratio and the resulting interface energy minimization [19]. In the measurement presented here, the current limitation from the serial resistance of 1 GΩ restricts the Ag mass transport, and consequently effectively narrows the filament growth. Thus, the experimental conditions under incorporation of the 1 GΩ serial resistance constrained the operation of the memtip in the diffusive switching regime. Generally, for a SET event, driving forces leading to interface energy minimization must be outweighed by the driving forces promoting filament formation, which originates from the external voltage. Accordingly, the RESET voltage indicates the operation regime where the driving forces for energy minimization cannot be compensated anymore. Both SET and RESET switching show a certain degree of randomness, and the respective threshold voltages underlay a statistical distribution. This is shown in Figure 2d, where 100 sequential cycles are depicted, showing that SET and RESET voltages span up a voltage interval.

**Figure 3:**
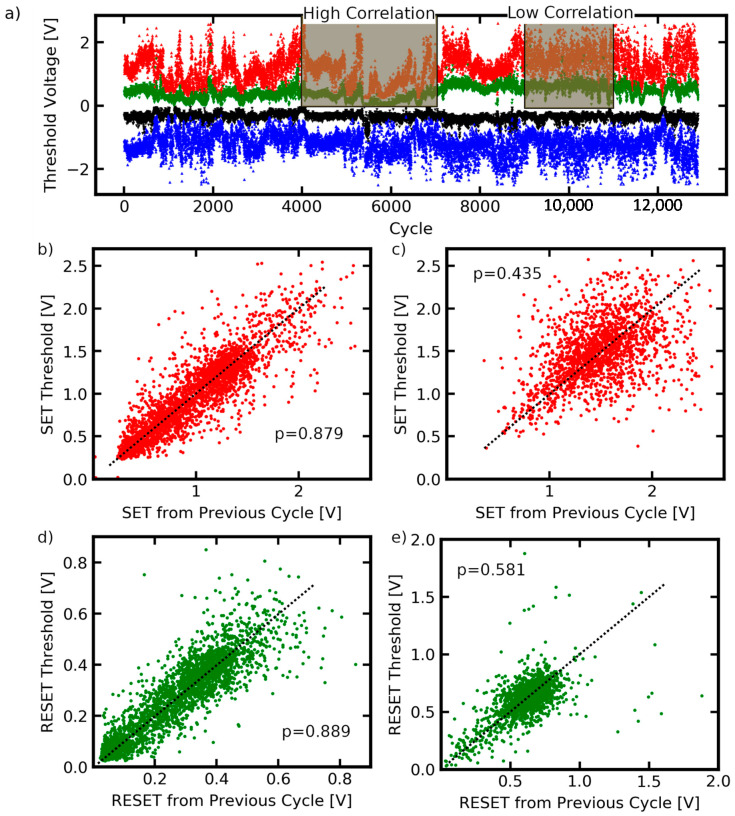
(**a**) All threshold voltages extracted from the long-term measurement ranging over 12,000 cycles of one memtip sample and plotted against the cycle number. In the indicated high correlation regime (cycles 4000 to 7000) the threshold voltages show a significant dependence on the former values from the previous cycles, resulting in pronounced trends in threshold voltage evolution over several cycles. In a later regime (cycles 9000 to 11,000), which suggests a low correlation, the individual threshold voltages are more independent from each other, and no pronounced trends are observable. The Pearson coefficient quantifies the linear correlation between the individual threshold voltages and the respective former value from the cycle before, for the SET and RESET events in the high correlation regime, (**b**,**d**), respectively, and the low correlation regime, (**c**,**e**), respectively.

Knowledge about the variability of switching parameters from cycle to cycle is of crucial importance for the application of memristive devices. Switching variability is an unwanted feature for certain applications like selector devices, because the state of each individual device has to be under precise control. However, the intrinsic randomness also offers promising approaches like probabilistic computing, or for hardware security applications, like true random number generators, where diffusive memristors are used as a source of uncertainty to create bit streams, which could be used as cryptographic keys [16]. Thus, basic research on the inherent variability of fundamental switching entities (like one single filament) is required to understand the implications on the device performance. However, the acquisition of reliable cycle-to-cycle variability data from switching phenomena on the nanoscale by conventional cAFM investigation is challenging because a large amount of sequential cycles must be measured to make meaningful statements, which is made impossible by thermal drift. Here, the long-term measurement of a memtip was used to gain deeper insights into the inherent variability of diffusive switching of one single filament. In Figure 3a, all threshold voltages are extracted and plotted against cycle number. A corresponding histogram plot and cumulative distribution function can be found in the Supporting Information Figures S4 and S5, respectively. Only cycles showing a clear diffusive switching characteristic, with a minimum switching window of 0.2 V were taken into account for this evaluation. A current level of 20 pA was consulted as criterion for threshold voltage detection, i.e., exceeding this level marks a SET event, and falling below this level marks a RESET event. Calculation of the variances (cf. Supporting Information Figure S4) for each threshold switching event indicated that the SET voltages (at both polarities) possess a more pronounced stochasticity than the RESET events. A notable observation is that the positions of threshold voltages are not stochastically independently distributed over the whole measurement, but the evolution of threshold voltages follows a common tendency over narrow intervals. This indicates a dependence on the history of device operation, and could be used to encode information in temporal correlations. To elucidate this, two regimes were highlighted. Systematic drifts of SET and RESET voltages at positive polarity governed the regime going from cycle 4000 to 7000. Separate drifts ranged over periods in the order of 100 cycles. In contrast to this, the threshold voltages in the regime from cycle 9000 to 11,000 occurred at a more constant level on average, and less occasional drifts were apparent. This suggests that individual SET and RESET voltages are not statistically independent from each other on compulsion, but correlated to the switching parameters from the previous cycles, and that the strength of correlation is an altering variable. Figure 3b,c plot the individual SET voltages at positive polarity against the respective values from the previous cycle for the first (cycles 4000 to 7000), and the second regime (cycles 9000 to 11,000), respectively, to illustrate the correlation strength. The same is shown for RESET voltages in Figure 3d,e. The Pearson coefficient was consulted as a measure to quantify the linear correlation between the pairs of values (i.e., the threshold voltage and the corresponding threshold voltage from the previous cycle) and given in the respective graphs. Regarding the first regime (cycle 4000 to 7000) the Pearson coefficient quantifying the correlation between SET events amounted to 0.879, and to 0.889 for RESET events. In the second regime (cycles 9000 to 11,000), the Pearson coefficient was calculated as 0.435 for SET events, and 0.581 for RESET events. Based on the quantification by the Pearson coefficient, the correlation between the individual threshold voltages and the respective former values from the previous cycle was stronger than in the first regime from cycle 4000 to 7000. A comparison of SET and RESET events within one cycle also reflects the higher degree of correlation in the regime from cycle 4000 to 7000 (cf. Supporting Information Figure S6). The appearance of correlation suggests the introduction of a type of autocorrelation function over the amount of cycles passed between the correlated cycles (cf. Supporting Information Figure S7). The fact that the degree of correlation reduces with a higher separation of the correlated cycles (in terms of number of cycles, which have been conducted in the interim period) is an important consideration for the design of filamentary diffusive switching devices, and could be used for bio-inspired information processing. The dependence of threshold voltages on the values from previous cycles becomes evident under consideration of the morphological configuration of the disintegrated filament after a RESET event. This is because the reconfiguration after the filament disintegration determines the preconditions for the subsequent SET switching, in terms of number of gaps between individual clusters and the corresponding separation lengths. As the electrical field is the decisive driving force for SET switching, any variation in the morphology of the disintegrated filament and its characteristic separation lengths affects the electrical field, and consequently the SET voltage for the following SET event. In the case that the disintegrated filament already shapes a pronounced preformed filament, SET switching is eased, and lower SET voltages are necessary. The subsequent RESET event will leave behind a disintegrated filament with similar morphological configuration. On the contrary, when the morphological configuration of the disintegrated filament exhibits poor filament preforming, SET switching is impeded, and higher SET voltages are required. The fact that the morphological configuration of the filament does not change drastically from cycle to cycle, but only over larger time scales, interprets the observed correlation between individual threshold voltages and the respective former value from the previous cycle. The observed alteration of the degree of correlation underlines the assumption that some morphological configurations of the filament bear more inherent randomness of the switching voltage than other configurations, and that the degree of correlation evolves in correspondence with the long-term change of the morphological configuration. Furthermore, it can be observed that the degree of correlation may also depend on the polarity of the external field. This is most prominent in the high correlation regime for SET and RESET at positive polarity (cycles 4000 to 7000). In this regime, the correlation coefficients for SET (0.574) and RESET (0.652) at negative polarity are significantly lower. This can be attributed to the geometrical asymmetry of the electrodes leading to different field distributions across the filament. The asymmetric field can cause polarity dependent switching kinetics with different degrees of correlation, because the number and morphology of anodic and catholic sites in the system can differ. These findings, facilitated by the memtip approach, indicate that correlations between sequential switching events are an essential feature of diffusive switching. Since the degree of correlation defines fundamental constraints on the applicability of diffusive switching devices, deeper understanding of this behavior is highly important.

### 3.2. Study of Switching Properties of an Identical Filament on a Gold and ITO Counter Electrode

One major issue for progressing with the reliable design of neuromorphic hardware is coping with different filament properties between different devices, that arise even though identical forming conditions were applied. Despite of great effort for achieving highly reproducible manufacturing of memristive devices, for example in a crossbar array architecture, there can be critical differences in the performance between individual devices within the hardware [25,26]. Critical differences affect fundamental parameters of the memristive device, like the SET and RESET voltages, or the forming and retention time of the state. A major source for such device-to-device variability is the necessity of an electroforming step in many memristive devices to initiate the first filament formation from the virgin state. Since precise control of the electroforming step is very challenging, and the morphology of the first evolved filament impacts on the device performance for all subsequent cycles, it is a major origin of the stochasticity between individual devices. Recent studies have shown that the defect state in the dielectric layer plays a crucial role in the switching kinetics [24]. Grain boundaries, for example, act as efficient conduction channels for Ag+-ions, and therefore assist the formation of a metallic filament in the dielectric layer. Differences in the defect state between individual memristive devices lead to different given conditions for the filament formation, and therefore to a different device performance. The variable filament properties in different devices not only complicate reaching the next level of hardware maturity, but also the search for the right analytical method, because it is always an unknown influence when the performance of different devices is compared. These difficulties have been encountered in several previous studies dedicated to the influence of the counter electrode in memristive devices [29,30,31].

Using the concept of memtips, IV hysteresis data on the impact of different counter electrodes can be obtained using the identical memtip, bringing it into reproducible electrical contact with different counter electrode materials in consecutive measurements. As an identical memtip is used, all conditions coming from the active electrode and dielectric layer, like defect structure or electroformed filament, are kept constant. The only varying parameter in this approach is the electrical contact between the memtip and counter electrode, which allows investigating the influence of this parameter with the same active filament. This approach is demonstrated in this section by the operation of an identical memtip on two different technologically relevant counter electrode materials, namely Au and ITO. We note, that such an approach is only meaningful for the purpose of basic research, and will not address the problem of filament variability with respect to practical applications. Furthermore, the application of memtips is only meaningful when data from an identical memtip is considered, as the variability between different memtips is expected to be high.

**Figure 4:**
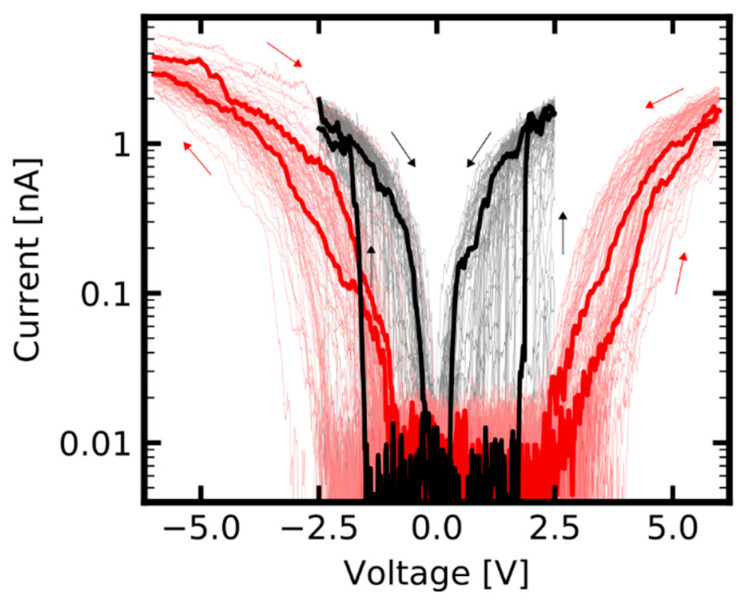
Electrical characteristic of an identical memtip operated over 50 cycles between −2.5 V and +2.5 V on an Au counter electrode (black cycles), and over 50 cycles between −6 V and +6 V on an ITO counter electrode (red cycles). Investigating an identical memtip allows the operation of the very same active filament on different counter electrode materials. For operation on ITO the switching window lies at higher voltages compared to operation on Au.

Figure 4 compares the switching characteristics of an identical memtip under operation on an Au, and an ITO, counter electrode. From this it becomes apparent that the switching characteristics of an identical memtip can be modified through variation of the memtip-to-counter-electrode contact. This can be most prominently described by a variation of the operation window, which is defined by the bias regime, where both HRS and LRS can be occupied. Regarding the highlighted representative cycle for Au in Figure 4, one can see that upon operation of the memtip on an Au counter electrode, the operation window in reference to the positive regime ranges from +0.3 V and +1.9 V. In comparison to this, the operation window on ITO, also in reference to the positive regime, is shifted to higher bias voltages above +2.5 V. A further important point is that the memtip can go into the LRS reliably when operated on Au for voltages between 0 V and +2.5 V, while on ITO only a HRS is observed in the same voltage range.

**Figure 5:**
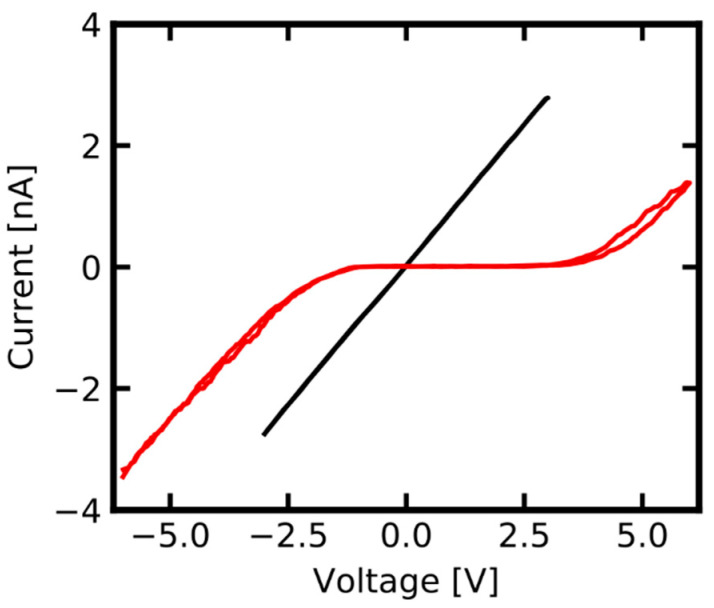
Comparison of the LRS of an identical memtip on an Au (black) and an ITO (red) counter electrode. For this representation, the medians of 10 sequential cycles on each electrode were taken which were consistently in LRS. The LRS on Au is clearly ohmic, and reflects the 1 GΩ serial resistance in the experimental setup. The LRS of the identical memtip on ITO is fundamentally different, and shows a non-linear behavior.

The good stability of the memtip approach enabled us to record a high number of cycles, with some cycles purely showing a LRS, i.e., no filament disintegration at low voltages. The sequences of such cycles, which purely show a LRS behavior, were evaluated in more detail to gain a deeper insight into the influence of different counter electrodes, and their consequences on device design, because those states reflect the most extensive filament formation and consequently the best contact between filament and counter electrode. The electrical characteristics during the LRS of the memtip on each of the two electrodes are shown in Figure 5. In the case of Au as the counter electrode, a clearly ohmic characteristic evolved when the memtip is in its conducting state, as is expected for memristive samples having an inert metal–dielectric medium–active metal structure. In the ohmic state, the overall resistance corresponds to the serial resistance in the cAFM instrumentation. Therefore, a deficient filament formation, or energetic barriers with corresponding rectifying properties of the contact between memtip and Au counter electrode, can be excluded as limiting mechanisms. However, upon operation of the identical memtip on an ITO counter electrode the conducting state became substantially different, since the electrical characteristics exhibited rectifying and asymmetric properties. Hence, the current was not limited solely by the 1 GΩ serial resistance anymore, but from an additional barrier arising from a Schottky contact between the memtip and the n-type ITO (cf. Supporting Information Figure S8).

### 3.3. Highly Dynamic HRS-LRS Transitions at the Border of Filament Stability

**Figure 6:**
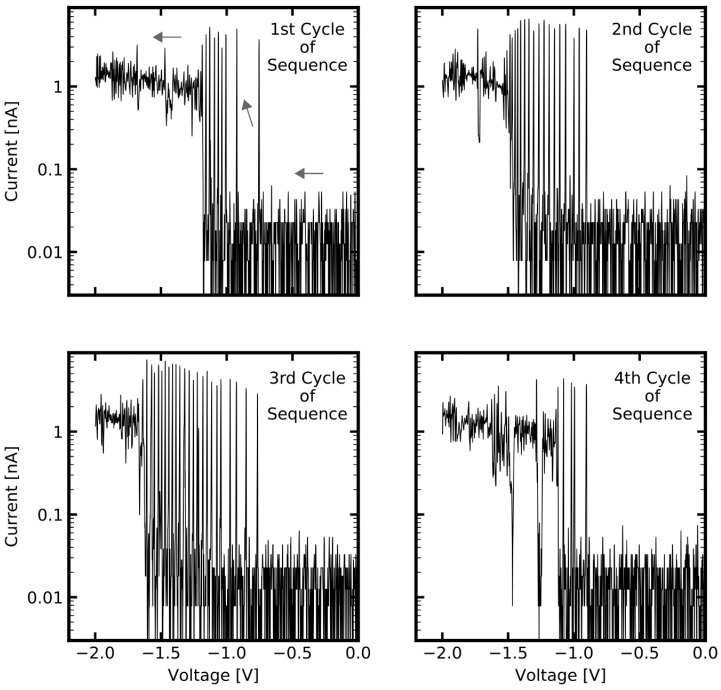
Highly dynamic HRS-LRS transitions, as observed when a memtip is operated at the border of filament stability. Shown are SET events at negative polarity extracted from four subsequent cycles. The arrows in the first panel indicate the direction of voltage sweep in these extracts (i.e., from zero towards 2 V). There is no persistent threshold switching but a dynamic behavior, arising from multiple attempts at filament formation followed by immediate disintegration.

This section focusses on features of diffusive memristive switching that emerge when a filamentary device is operated under conditions where persistent transitions between HRS and LRS are impeded. The operation under such conditions is characterized by multiple back and forth switching between HRS and LRS, leading to highly dynamic switching patterns, as shown in Figure 6.

**Figure 7:**
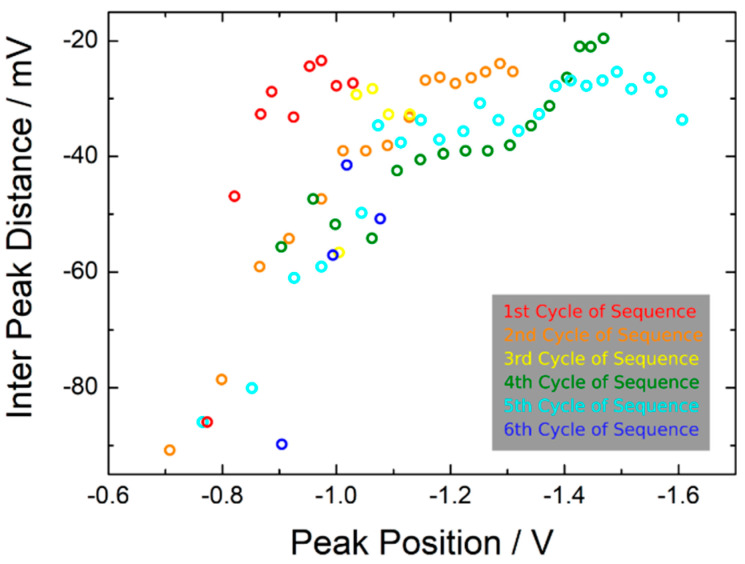
Data of highly dynamic SET switching patterns at negative polarity from six consecutive cycles evaluated with a view on interspike intervals (ISIs). The ISIs from the 3rd to 6th cycle of the given sequence correspond to the highly dynamic SET switching patterns presented in Figure 6. One can see that the ISIs decrease (i.e., the number of switching attempts increases) with increasing stimulation from the external voltage ramp (i.e., peak position).

The memtips which were discussed in Section 2.1 and Section 2.2 were fabricated with effective thicknesses of 3.5 nm for Ag, and 4 nm for Si_3_N_4_ and exhibited stable diffusive switching in roughly 95% of the cycles. In particular, it was seen that the SET switching events were persistent, meaning that a following RESET event must be triggered by decreasing the voltage close to zero volts. In contrast, the memtip discussed in this section had an effective thicknesses of 2 nm for Ag, and 6 nm for Si_3_N_4_, i.e., the reservoir for mobile Ag+-cations is more limited, and the minimum filament length in order to bridge the dielectric layer is enlarged. From the electrical characterization of the memtip with 2 nm Ag and 6 nm Si_3_N_4_ on an Au counter electrode by IV-sweeps from −2 V to +2 V, we found a drastically lower yield of cycles exhibiting clear diffusive switching, of about 22%. The residual 78% of all cycles showed persistent occupation of the HRS, or only occasional switching events. This indicates that stable filament formation was impeded in this memtip under the given experimental conditions. Among all cycles, a considerable amount of switching events exhibited pronounced highly dynamic HRS–LRS transitions prior to occupation of the LRS. The presented highly dynamic HRS–LRS transitions in Figure 6 were extracted from the SET switching events at negative polarity (i.e., in these extracts the voltage is ramped from zero to −2 V) of four subsequent cycles. One can see that there are various attempts at SET switching, which are, however, directly abandoned. Furthermore, it can be seen that the frequency of the incomplete SET switching attempts gets higher with increasing absolute voltage. This can be clearly seen, for instance in the first cycle of the sequence, where the first and second switching attempts that occur are more isolated, followed by a burst-like sequence of six switching attempts, before the memtip finally switched into the LRS. To confirm that the observed behavior arises from the Ag filament dynamics, we performed a reference measurement with a memtip prepared without Ag layer, showing no indication of memristive switching (cf. Supporting Information Figure S9). Such a behavior closely resembles the spiking patterns in neural coding schemes [32]. A view of interspike intervals, which is an essential characteristic in neural coding, for the observed dynamic patterns can be found in Figure 7. The evaluation of interspike intervals underlines the observation that the frequency of switching attempts increases with increasing absolute voltage.

It can be supposed, that such dynamic patterns occur at the border of filament stability because a distinct occupation of either HRS or LRS is impeded in a narrow voltage interval, as observed. One can argue that filament instability is induced in this memtip by increasing the thickness of Si_3_N_4_ and more limiting the Ag reservoir (in comparison to the memtips in Section 2.1 and Section 2.2), while keeping the magnitude of current flowing through the device during operation constant. This can be understood from the Rayleigh instability, which is widely claimed to be responsible for spontaneous filament disintegration [19,33,34]. Accordingly, spontaneous disintegration is energetically favored in case of a thinner filament diameter, and kinetically accelerated by the number of perturbations at the filament. The main influence on the filament diameter, which is the current limitation, was kept constant throughout this whole work. However, the probability for perturbations, which scale with the length of the filament, leading to acceleration of filament disintegration, is increased because of the longer filament which is required to override a thicker dielectric layer.

In combination with the induced filament instability, the occurrence of highly dynamic HRS–LRS transitions can be understood from the fact that the driving force promoting the filament formation is given by the external potential and immediately drops at the moment of SET switching, because of the formed conductive path connecting both electrodes. Here, the serial resistor implemented in the experimental setup plays a crucial role: As long as the memtip is in its LRS, the majority of external potential drops over the serial resistance. If a critical diameter for filament stability is not reached at that point, the filament immediately disintegrates due to interface energy minimization, and the LRS will not be maintained. This, in turn, is accompanied by an increase of the external potential which triggers the next switching attempt, until a persistent filament has been formed. Those described counteracting driving forces play a fundamental role in many filamentary diffusive memristive switching devices. Based on the assumption that the interplay of those counteracting driving forces is essentially responsible for the observed spiking behavior, one can argue that such spiking dynamics can be expected in most filamentary diffusive switching devices.

Regarding applications, the design and investigation of devices which are able to transfer electrical signal inputs into a spiking pattern is of great interest in the field of neuromorphic engineering. From this, memristive systems with short-term memory behavior can be derived, which could play an important role in the hardware-based processing of sensory data [35,36,37]. The utilization of memtips turned out to be advantageous for observations at the border of filament stability and related spiking behavior, because constraining the action of the filament to the apex through direct integration of the memristive device provides the most reliable contact between probe and filament.

## 4. Conclusions and Outlook

In this work, an unconventional cAFM approach [27] was adapted, which provided us enhanced capabilities for the electrical characterization of ECM memristive devices on the nanoscale. This approach intended to integrate the memristive device of interest directly on the apex of a conductive cantilever instead of conventional probing. By contact establishment between the functionalized cantilever to any kind of counter electrode material, electrical characterization is done via regular cAFM instrumentation. Considerable advantages emerge from such an approach, such as mitigation of thermal drift effects during long-term measurements, because the active area for switching is constrained to the apex of the cantilever. Furthermore, it enables measurement strategies for testing the same active filament on different counter electrodes. Along with the demonstration of this approach for ECM devices, fundamental insights into the diffusive switching performance of an Ag/Si_3_N_4_ stack, which is an archetypical filamentary memristive device, were gained. The stability of the memtip approach facilitated a long-term measurement over 12,000 consecutive cycles. From this, regimes with different degrees of temporal correlation between subsequent switching events were identified during the long-term operation. Moreover, an energetic barrier between the Ag filament and an ITO counter electrode was identified, by comparing the IV characteristics of an identical memtip in electrical contact to an Au and an ITO counter electrode, respectively. Finally, highly dynamic HRS–LRS transitions were observed, which presumably occur at the border of filament stability. This work shows, that investigations on ECM devices using memtips are a reliable and powerful tool for gaining insights into the performance of memristive devices, and provide experimental possibilities that go beyond conventional cAFM. This approach can be easily extended towards various materials systems and contact scenarios, and offers the potential to gain a deeper understanding of memristive switching on the nanoscale.

## Data Availability

The data presented in this study are available on request from the corresponding author.

## Supplementary Materials

The following are available online at https://www.mdpi.com/2079-4991/11/2/265/s1, Figure S1: Thickness estimation of the Si_3_N_4_ matrix from the TEM micrograph. Figure S2: Topographic AFM images acquired with a memtip. Figure S3: TEM micrographs before and after prolonged TEM investigation on memtips. Figure S4: Histogram plot representation of threshold voltages extracted from the long-term measurement. Figure S5: Cumulative distribution function of threshold voltages extracted from the long-term measurement. Figure S6: Correlation between SET and RESET events. Figure S7: Pearson coefficient versus separation of the correlated cycles. Figure S8: Band diagram discussing the energetic barrier between Ag and ITO. Figure S9: Reference measurement from a memristive cantilever sample reproduced without active Ag layer.
