# Supplementary material for: Enhancing Reliability of Studies on Single Filament Memristive Switching via an Unconventional cAFM Approach
Source: Nanomaterials (Basel). 2021 Jan 20;11(2):265. doi: 10.3390/nano11020265 (PMC7909531; doi:10.3390/nano11020265)
Supplement: Supplementary file 1 [file nanomaterials-11-00265-s001.pdf]

Supporting Information

Enhancing Reliability of Studies on Single Filament Memristive Switching via an Unconventional cAFM Approach

Niko Carstens, Alexander Vahl, Ole Gronenberg, Thomas Strunskus, Lorenz Kienle, Franz Faupel *, and Abdou Hassanien *

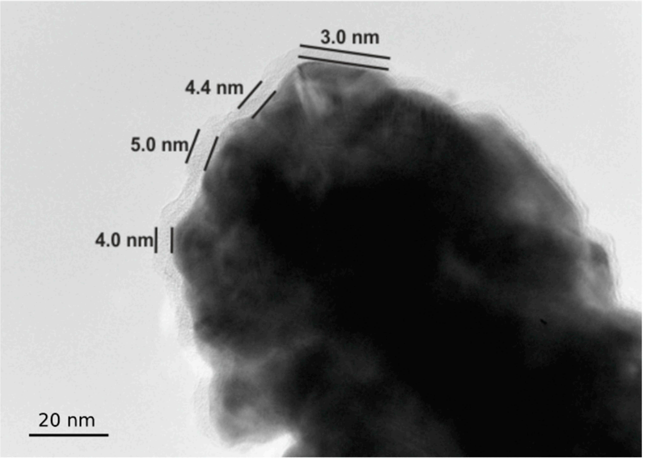

Figure S1. Thickness estimation of the Si_3N_4 matrix from the TEM micrograph.

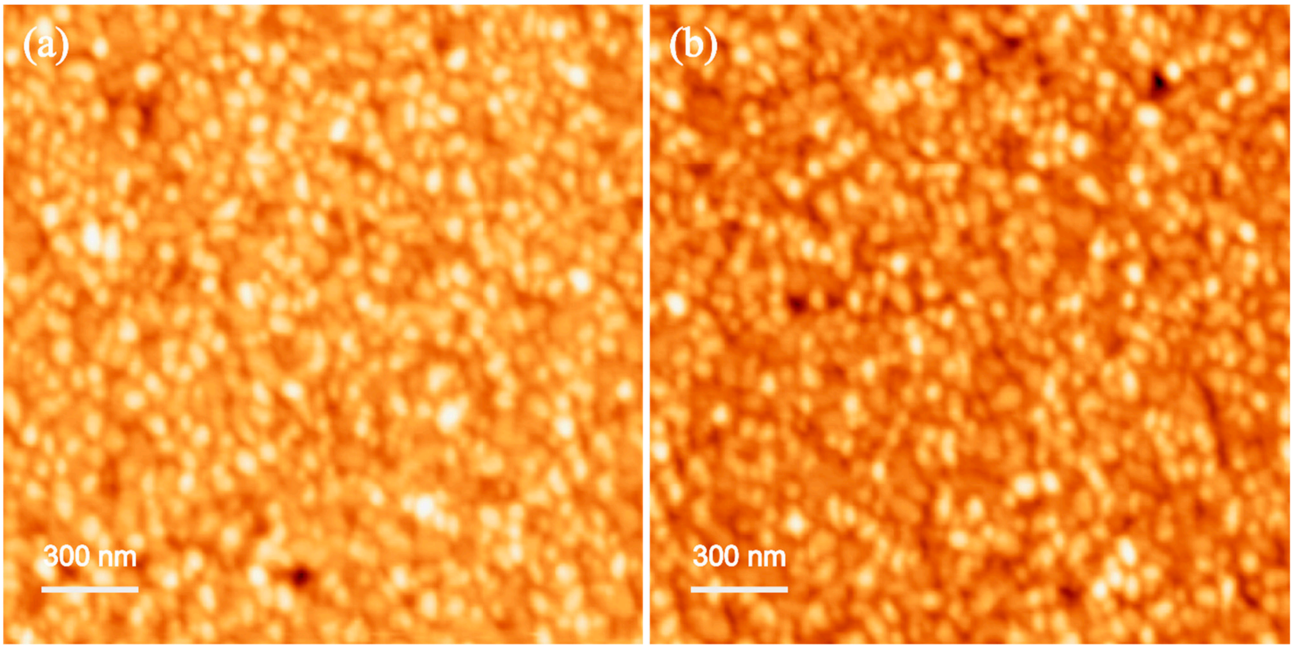

Figure S2. Topographic AFM images in tapping mode showing the structure of Au electrode imaged by memristive cantilever sample before (a) and after (b) prolonged IV measurements with a maximum current set to a few nA. Small

features of less than 20 nm can be easily identified in both images which indicate that the tip apex remained intact during measurements.

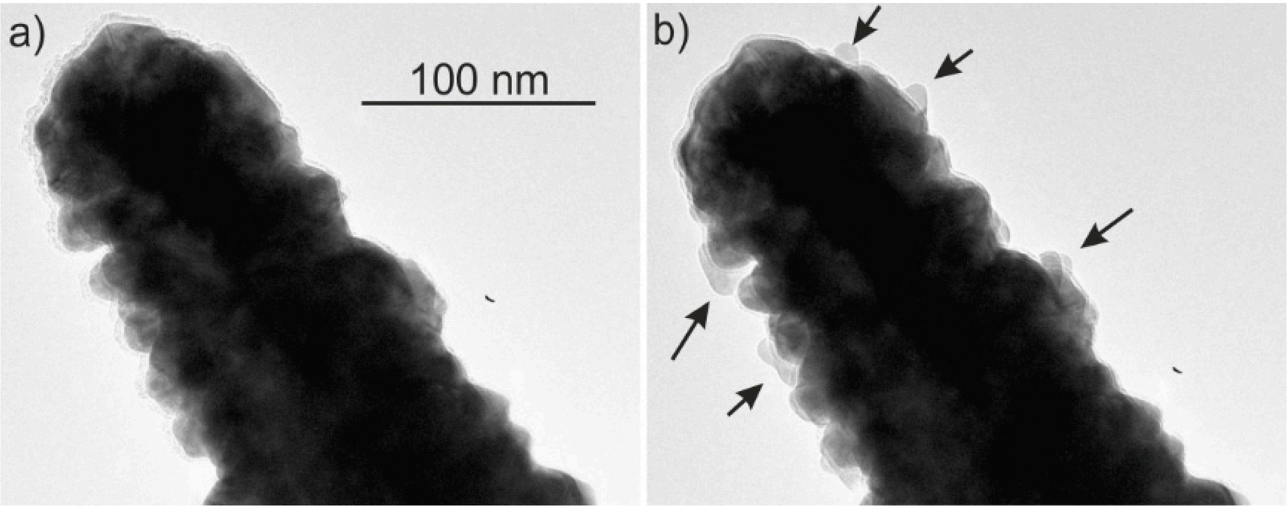

Figure S3. TEM micrographs before (a) and after (b) prolonged TEM investigation. Electron beam irradiation during TEM investigation of memristive cantilever samples causing morphological changes. After prolonged investigation Ag protrusions (marked by the arrows) are forming. Further, the dielectric matrix changes its morphology from rough to thinner and smooth. The insulating behavior is most likely due to the migration of the Ag from the apex to the protrusions so that not sufficient active Ag remains at the apex.

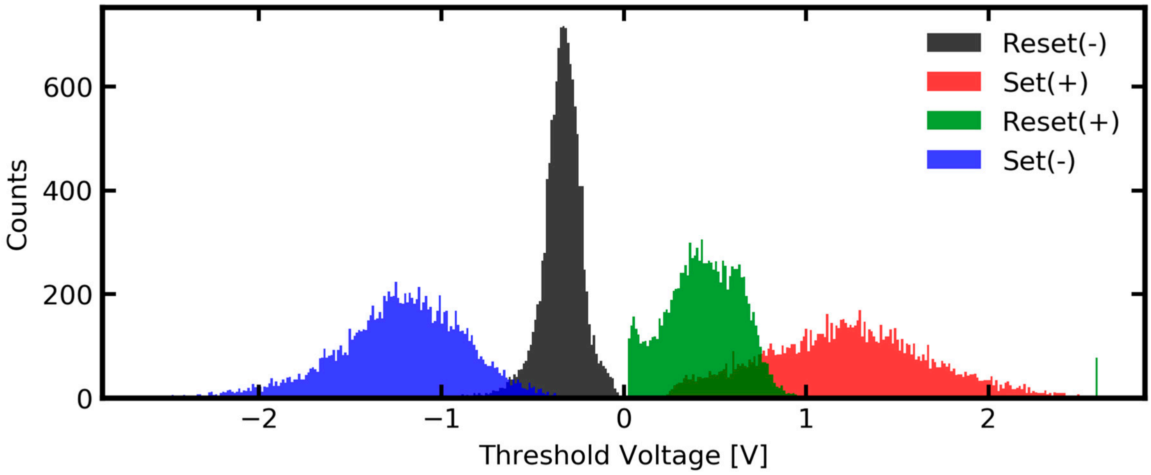

Figure S4. Histogram plot representation of threshold voltages extracted from the long-term measurement (cf. figure 3a in main text). The variances of the distributions can be calculated as 0.0132 for Reset(-), 0.2072 for SET(+), 0.0435 for RESET(+) and 0.1245 for SET(-).

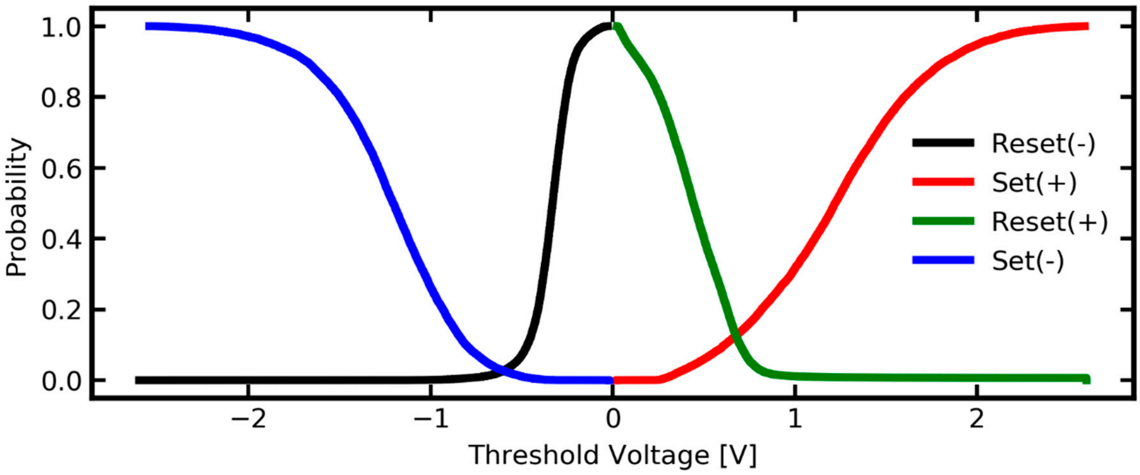

Figure S5. Cumulative distribution function of threshold voltages extracted from the long-term measurement (cf. figure 3a in main text).

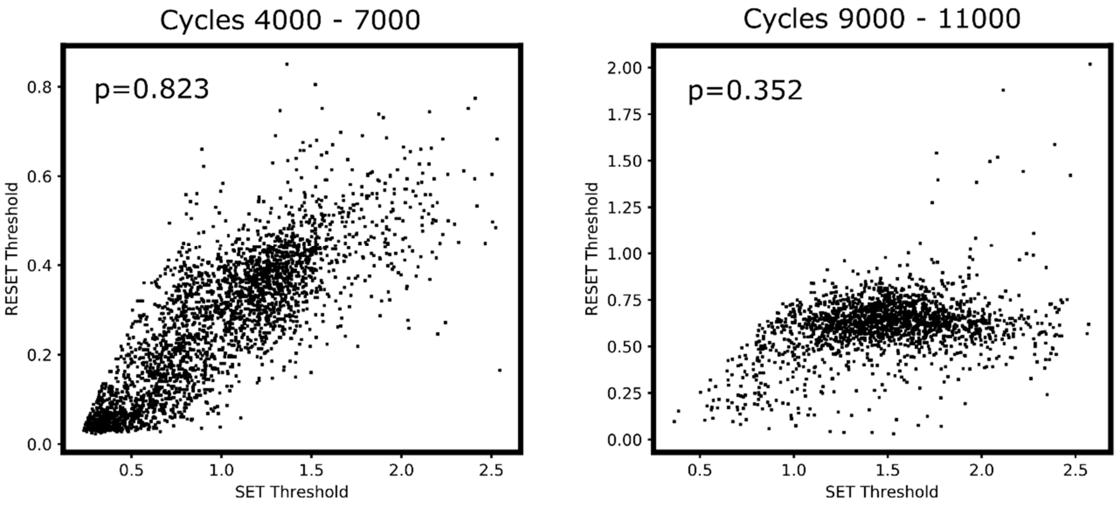

Figure S6. Correlation of SET and following RESET voltages for the period between cycles 4000 to 7000 and 9000 to 11000. It can be seen that the switching in the first period is clearly correlated, whereas it is more random in the second period.

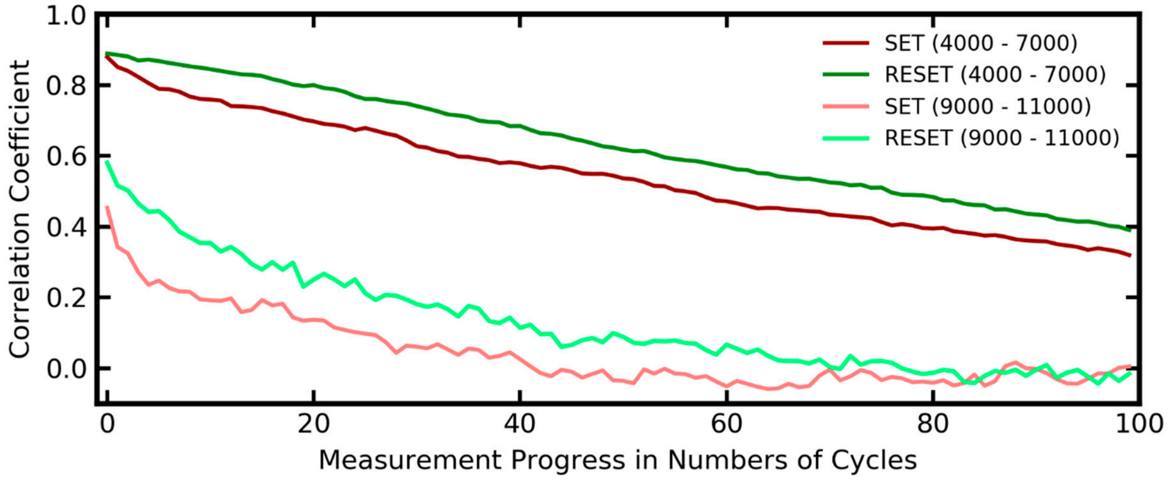

Figure S7. Pearson coefficient versus separation of the correlated cycles (separation is given in number of cycles which are conducted between the cycles). The more cycles were conducted in between the lower the correlation becomes. SET and RESET events in the region from cycle 4000 to 7000 show also consistently a stronger a correlation than in the region from cycle 9000 to 11000 when the correlated cycles are zero to 100 conducted cycles apart from each other.

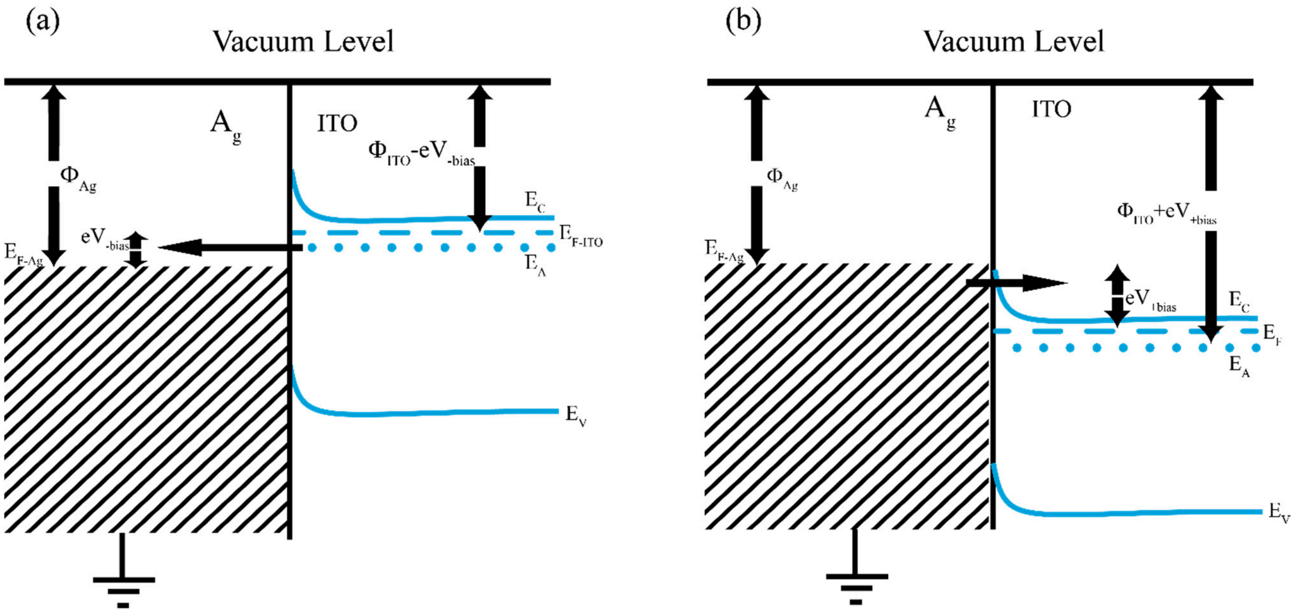

Figure S8. Schematic illustration of energy band diagram of Ag filament and ITO substrate. The rectifying behavior arises from Schottky contact where electrons flow easier when ITO is negatively biased as in (a) than positively biased as in (b).

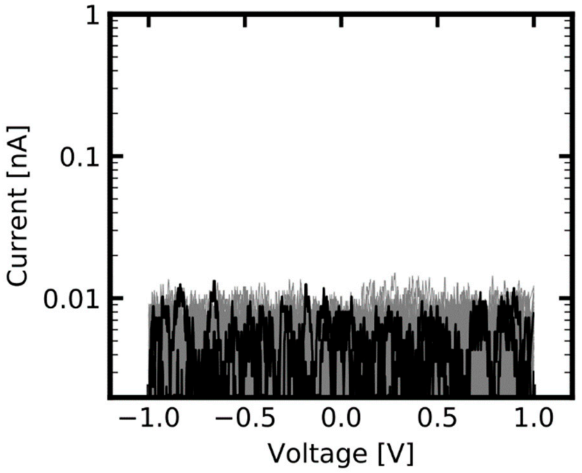

Figure S9. Reference measurement (40 cycles) from a memristive cantilever sample reproduced without active Ag layer.

There are no indications on switching events, which approves that the memristive action originates from the Ag layer.
